# Crystal Structures of TbCatB and Rhodesain, Potential Chemotherapeutic Targets and Major Cysteine Proteases of *Trypanosoma brucei*

**DOI:** 10.1371/journal.pntd.0000701

**Published:** 2010-06-08

**Authors:** Iain D. Kerr, Peng Wu, Rachael Marion-Tsukamaki, Zachary B. Mackey, Linda S. Brinen

**Affiliations:** 1 Department of Cellular and Molecular Pharmacology, University of California San Francisco, San Francisco, California, United States of America; 2 Department of Pathology and the Sandler Center for Basic Research in Parasitic Diseases, University of California San Francisco, San Francisco, California, United States of America; Yale School of Public Health, United States of America

## Abstract

**Background:**

*Trypanosoma brucei* is the etiological agent of Human African Trypanosomiasis, an endemic parasitic disease of sub-Saharan Africa. TbCatB and rhodesain are the sole Clan CA papain-like cysteine proteases produced by the parasite during infection of the mammalian host and are implicated in the progression of disease. Of considerable interest is the exploration of these two enzymes as targets for cysteine protease inhibitors that are effective against *T. brucei*.

**Methods and Findings:**

We have determined, by X-ray crystallography, the first reported structure of TbCatB in complex with the cathepsin B selective inhibitor CA074. In addition we report the structure of rhodesain in complex with the vinyl-sulfone K11002.

**Conclusions:**

The mature domain of our TbCat•CA074 structure contains unique features for a cathepsin B-like enzyme including an elongated N-terminus extending 16 residues past the predicted maturation cleavage site. N-terminal Edman sequencing reveals an even longer extension than is observed amongst the ordered portions of the crystal structure. The TbCat•CA074 structure confirms that the occluding loop, which is an essential part of the substrate-binding site, creates a larger prime side pocket in the active site cleft than is found in mammalian cathepsin B-small molecule structures. Our data further highlight enhanced flexibility in the occluding loop main chain and structural deviations from mammalian cathepsin B enzymes that may affect activity and inhibitor design. Comparisons with the rhodesain•K11002 structure highlight key differences that may impact the design of cysteine protease inhibitors as anti-trypanosomal drugs.

## Author Summary

Proteases are ubiquitous in all forms of life and catalyze the enzymatic degradation of proteins. These enzymes regulate and coordinate a vast number of cellular processes and are therefore essential to many organisms. While serine proteases dominate in mammals, parasitic organisms commonly rely on cysteine proteases of the Clan CA family throughout their lifecycle. Clan CA cysteine proteases are therefore regarded as promising targets for the selective design of drugs to treat parasitic diseases, such as Human African Trypanosomiasis caused by *Trypanosoma brucei*. The genomes of kinetoplastids such as *Trypanosoma spp.* and *Leishmania* spp. encode two Clan CA C1 family cysteine proteases and in *T. brucei* these are represented by rhodesain and TbCatB. We have determined three-dimensional structures of these two enzymes as part of our ongoing efforts to synthesize more effective anti-trypanosomal drugs.

## Introduction

The protozoan parasite *Trypanosoma brucei* is the cause of Human African Trypanosomiasis (HAT, sleeping sickness) in humans and nagana in domestic livestock [1], [2], [3]. With over 60 million people at risk and 50,000–70,000 infected, new drugs are required to control the spread of disease and associated mortality. Only four drugs are approved for treatment [4], however, most are limited by parasite resistance [4] and marked host toxicity [5], [6], [7], [8]. A new combination therapy for treating HAT has been approved recently by the World Health Organization (WHO), however no newly developed drugs are on the horizon [9], [10]

Clan CA cysteine proteases play central roles during the lifecycle of many parasitic organisms [11] and have been established as effective drug targets in treating many parasitic diseases [12], [13], [14], [15]. Bloodstream *T. brucei* parasites express two papain family cysteine proteases, rhodesain (brucipain, trypanopain), a cathepsin L-like enzyme and TbCatB, a cathepsin B-like enzyme. Rhodesain is the more abundant of the two cathepsins and is required to cross the blood-brain barrier [16]. RNA interference of TbCatB is able to rescue mice from a lethal *T. brucei* infection [16]. RNAi knockdown of rhodesain, however, only prolongs mouse survival [17]. TbCatB may therefore represent the more promising target for novel cysteine protease inhibitors targeting *T. brucei* infection. A cysteine protease inhibitor, Z-Phe-Ala-CHN2, has been shown to be lethal to *T. brucei* both *in vitro* and *in vivo* [18], [19] and our efforts are focused on elucidating key features of inhibitors that will optimize both specificity and potency.

Both cathepsin B-like and cathepsin L-like proteases share the common features of Clan CA cysteine proteases, including a conserved catalytic triad (Cys/His/Asn) and a substrate-binding site comprised of many structurally conserved residues [20]. One major difference between cathepsin L and cathepsin B enzymes is the presence of an ‘occluding loop’ of approximately 20 amino acids located on the surface of cathepsin Bs that confers an additional exopeptidase activity to these cysteine proteases [21].

To elaborate on the structural and biochemical differences between cathepsin L and cathepsin B-like cysteine proteases in *T. brucei*, and to aid in the design of better inhibitors, we have determined the high-resolution crystal structures of rhodesain•K11002 and the first crystal structure of *T. brucei* cathepsin B, TbCatB•CA074.

## Materials and Methods

### Production of the TbCatB N-glycosylation mutant

Recombinant TbCatB was modified from a previously described protocol [22]. The gene encoding the full-length zymogen (minus the N-terminal signal sequence) was sub-cloned from a pPICZαB-TbCatB construct into the pPICZαA expression vector (Invitrogen). Site-directed mutagenesis using the QuickChange system (Stratagene) was used to add a C-terminal His tag and to incorporate an N216D mutation (full-length numbering) at a predicted glycosylation site. The expression of a glycoslylation site mutant in *P. pastoris* is a strategy that has recently been successful in our structural studies of the homologous parasite cysteine protease cruzain [23], [24].

### Expression and purification of TbCatB

*Pichia pastoris* strain ×33 was transformed with 20µg of BstXI linearized TbCatB-N216D according to the manufacturer's instructions (Invitrogen). A single transformed colony was used to inoculate 2–3 ml of YPD-zeocin media and the culture was grown overnight at 30°C. The following day, 2 liters of YPD-zeocin media were inoculated with the starter culture and incubated at 30°C, with constant shaking at 250rpm, until cell density reached an OD_600_ of 3–4 (typically 2–3 days). Cells were harvested at 1500g for 15min and the pellet was rinsed with 100ml BMM media and centrifuged at 1500g for 15min to remove residual YPD media. Cells were then harvested and resuspended in BMM media to an OD_600_ of approximately 1.0. Induction of protein overexpression was carried out in a BioFlo110 Fermentor/Bioreactor (New Brunswick Scientific) with the addition of 1% methanol, twice a day. Supernatant was collected after 3 days incubation and concentrated to 50ml using an Ultrasette™ lab tangential flow device with a 30kDa cut-off (Pall Corporation). The concentrated sample was adjusted to a final concentration of 300mM NaCl and 10mM Imidazole and incubated with 2ml of Ni-NTA beads (Qiagen) overnight at 4°C. The beads with the bound sample were transferred to an empty PD-10 column (GE Healthcare), rinsed with 100mM phosphate pH 6.0, 300mM NaCl, 10mM Imidazole, and eluted with 100mM phosphate pH 6.0, 300mM NaCl and 200mM Imidazole. Eluted proteins were dialyzed against 1L buffer containing 20mM Tris-HCl pH 8.0, 1mM EDTA and 5mM β-mercaptoethanol. Dialysis buffer was changed after 2 hours and continued overnight at 4°C.

### Activation, inhibition and purification of TbCatB

To produce the mature form of the protease, purified enzyme was auto-activated in a buffer containing 100mM sodium acetate pH 4.5, 10mM DTT, 1mM EDTA, 100mM NaCl and 100ug/ml dextran sulfate (MW 5000). Protease activity was monitored every hour using Z-Phe-Arg-AMC as the substrate [22], [25], [26]. After reaching its maximum, activity was completely abolished with the addition of 10-fold molar excess CA-074 (Sigma-Aldrich). The mixture was incubated overnight, with gentle stirring, to ensure complete inhibition of the activated enzyme.

Inhibited enzyme was then dialyzed against 20mM Tris-HCl pH8.0, 1mM EDTA and 5mM β-mercaptoethanol and applied to a Mono-Q column (GE Healthcare) with TbCatB•CA074 eluting at approximately 150mM NaCl (gradient 0–1M NaCl) at a flow rate of 1ml/min. Fractions corresponding to the protease were pooled and further purified on a Superdex 200 gel-filtration column (GE Healthcare). The purified sample was concentrated to 3mg/ml and the buffer exchanged to 20mM Tris-HCl pH 8.0.

### Edman sequencing of activated TbCatB

Three separate aliquots of TbCatB from the same batch were activated, purified and inhibited with CA074 as described above. 10–20µg of activated, inhibited protein was run on an SDS-PAGE gel and transferred on to a PVDF membrane. The blot was run for 90mins hrs at a current of 125mA. The same experiment was performed with a single purified, unactivated, sample of the enzyme. Bands containing mature and full-length TbCatB were excised from the membrane and sent for N-terminal Edman sequencing at the Protein and Nucleic Acid Facility (PAN), Stanford University Medical Center (http://cmgm.stanford.edu/pan/).

### Western Blot analysis of recombinant and native TbCatB

*T. brucei* were cultured in HMI-9 medium to a density of approximately 1.5×10^6^ tryps per/ml. To obtain crude extracts, tryps were pelleted in 50 ml conical centrifuge tubes by centrifugation at 2500 rpm. The medium was aspirated and the pellet re-suspended in 250 of lysis buffer (50mM Sodium Acetate pH 5.5, 1mM EDTA, 1% Tx-100). The lysate was clarified by centrifugation and the protein concentration of the supernatant was measured by Bradford assay (Bio-Rad). Recombinant, activated TbCatB was prepared and purified to the stage of anion exchange chromatography (Mono Q), as above. The crude lysate containing native TbCatB and the activated recombinant sample were both prepared for analysis by adding 5× SDS loading buffer and boiling for 2-minutes. 40 µg of the crude lysate or 0.3µg of purified activate TbCatB was loaded into the well of a Novex 12-well Bis-Tris mini gel (Invitrogen) and resolved by SDS-PAGE at 180 volt with constant current for 1.5 hours. The gel was transferred onto a PVDF membrane (Bio-Rad) and blocked for 2 hours in buffer containing 3% milk and 0.5% BSA. After blocking, the blots were incubated with rabbit anti-TbCatB antiserum and diluted 1∶1,000 overnight at 4°C. The blots were washed 3 times for 5 minutes with TBST and then incubated at room temperature for 1 hour with goat anti-rabbit serum (GE Healthcare) diluted at 1∶1,000 in TBS. Afterwards, the blots were washed 3 times for 5 minutes with TBST and once with TBS. The immunoblots were then analyzed by ECL reagent (GE Healthcare) (Figure S1).

### Crystallization of recombinant TbCatB•CA074

Crystallization conditions were screened with a Mosquito drop-setting system (TTP Labtech) against a number of commercially available kits. Optimization of crystal conditions was performed manually on the basis of initial screening hits. Hanging drops of 1–2µl were set up with 3mg/ml TbCatB•CA074, and 1M LiCl, 10% PEG 3350, 0.2M Tris-HCl pH 7.6. Rod-shaped crystals formed after 3 days and reached a maximum size after 5–10 days. Crystals were flash-cooled in well solution supplemented with 30% glycerol and mounted for the Stanford Auto Mounter (SAM) system [27].

### Crystallization of recombinant rhodesain•K11002

Rhodesain was expressed in *P. pastoris* and purified and activated as described previously [25], [26], [28] with a Ser172Ala mutation incorporated to remove an N-glycosylation site from the mature domain of rhodesain. Active rhodesain was incubated with a 10-fold molar excess of the inhibitor K11002, dissolved in DMSO. Complete inhibition of enzymatic activity was confirmed by fluorometric assay against the substrate Z-Phe-Arg-Nmec (Bachem). Purified rhodesain was concentrated to approximately 8 mg/ml in preparation for crystallization. Crystals of maximum size were obtained after approximately 10 days *via* the sitting drop method, from a precipitating solution of 1.6M ammonium sulfate, 0.1M Bicine pH 9.0 at 18°C. Crystals were flash-cooled in liquid nitrogen in well solution supplemented with 20% ethylene glycol.

### Structure determination of TbCatB•CA074 and rhodesain•K11002

All diffraction data were collected at the Stanford Synchrotron Radiation Lightsource (SSRL). Rhodesain•K11002 data were collected to 1.16Å on BL9-1 after selecting an optimal crystal from screening performed with the robotic SAM system [27]. TbCatB•CA074 data were collected following a similar protocol on SSRL BL7-1 with the best crystals diffracting to 1.6A resolution. For both datasets, reflections were indexed and integrated in MOSFLM [29] and scaled and merged in SCALA [30]. The TbCatB•CA074 structure was solved by molecular replacement using MOLREP [31] with a homology model built by MODELLER [32] from the ensemble coordinates of human cathepsin B (1GMY), rat cathepsin B (1CTE) and cruzain (1F2A). Two clear rotation function solutions were obtained in space group P2_1_ with peak heights/sigma of 13.27 and 12.91 respectively, corresponding to two molecules in the asymmetric unit. The translation function yielded a clear solution for the dimer with a score of 0.53 and initial R_factor_ of 54.6%. The structure of rhodesain•K11002 was solved by molecular replacement using PHASER [33] with a model derived from a prior structure of rhodesain bound to a different vinyl sulfone containing inhibitor (PDB ID 2P7U). A single, strong solution was obtained in space group P2_1_2_1_2_1_ with a rotation function Z-score of 19.3, a translation Z-score of 28.7 and an initial LLG (log likelihood gain) of +896, which improved to +2022.7 with 6 cycles of rigid body refinement.

Following rigid body and maximum likelihood restrained refinement in REFMAC5 [34], the inhibitor molecules were placed in clear mFo-DFc difference electron density in using COOT [35]. During these initial stages of refinement the occluding loop residues in TbCatB were removed from the model and rebuilt as the difference density became clear. Both models were completed through iterative rounds of manual model building and refinement with COOT [35] and REFMAC5 [34]. TLS parameterization was used to refine the TbCatB•CA074 structure, while anisotropic temperature factors were refined in the case of rhodesain•K11002. Water molecules were placed in each structure with COOT and manually assessed. The final rhodesain•K11002 model contains 1 molecule of rhodesain, 1 inhibitor molecule, 402 water molecules and 11 ethylene glycol molecules. The final TbCatB•CA074 model contains 2 molecules of TbCatB, 2 inhibitor molecules, 572 water molecules, 6 glycerol molecules, 1 Tris molecule, a lithium ion and a magnesium ion. Statistics for data collection and refinement are given in Table 1. The coordinates and observed structure factors amplitudes for rhodesain•K11002 and TbCatB•CA074 have been deposited in the Protein Data Bank under accession codes 2P86 and 3HHI respectively.

**Table 1:** X-ray data collection and refinement statistics.

Data Collection	rhodesain•K11002	TbCatB•CA074
Resolution	1.16 (1.19-1.16)	1.60 (1.69-1.60)
Space Group	P2_1_2_1_2_1_	P2_1_
Unit cell parameters		
a, b, c (Å)	33.66, 78.63, 80.73	53.88, 75.13, 75.90
α, β, γ (°)	90.0, 90.0, 90.0	90.0, 105.0, 90.0
Total unique reflections	68633	74519
Completeness	91.9 (80.0)	96.9 (95.5)
Redundancy	7.1 (6.9)	9.5 (9.5)
R_merge_	0.043 (0.120)	0.093 (0.687)
I/σ I	27.2 (14.9)	17.6 (4.8)
Wilson B-factor (Å^2^)	6.1	15.1

Refinement		
Resolution range (Å)	40.36–1.16	73.32-1.60
R_free_ (%)	13.0	17.8
R_factor_ (%)	11.0	14.7
Number of water molecules	447	572
Average B factor (Å^2^)		
protein	8.51	10.69
water	24.0	23.32
Rms deviations		
Bond lengths (Å)	0.017	0.020
Bond angles (°)	1.88	1.72
Ramachandran plot^1^		
Residues in favored regions (%)	95.9	96.7
Residues in allowed regions (%)	99.1	100
Number of outliers	2	0
PDB ID	2P86	3HHI

1 as defined by Molprobity [46].

## Results

### Overall structures of rhodesain•K11002 and TbCatB•CA074

**Figure 1:**
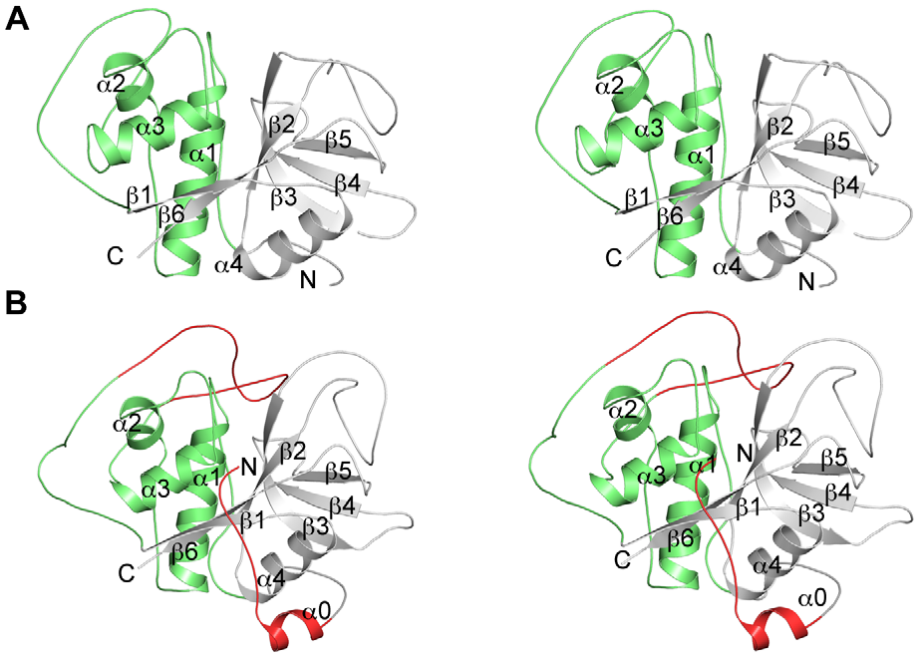
Structures of rhodesain and TbCatB. Stereo pairs of (A) rhodesain and (B) TbCatB. The figure is annotated with the secondary structure and the L and R domains are colored green and grey respectively. The occluding loop and N-terminal extension in TbCatB are colored red.

**Figure 2:**
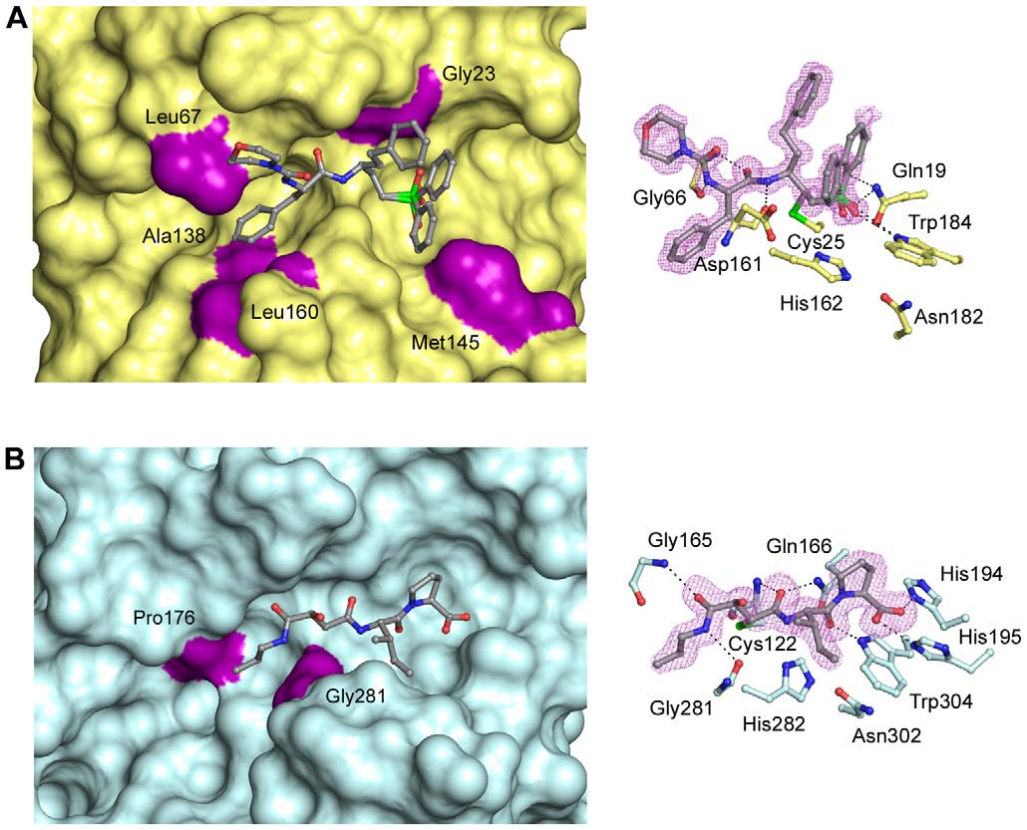
Inhibitor binding in TbCatB and rhodesain. Surface (left) and ball and stick (right) active site representations of (A) the rhodesain•K11002 complex and (B) the TbCatB•CA074 complex. Inhibitor molecules are colored grey and the unbiased mFo-DFc electron density for each is colored violet. Hydrophobic interactions with neutral/non-polar residues are mapped on the surface and colored purple.

The structure of rhodesain•K11002 was determined to 1.16Å resolution and refined to an R_free_ of 13.0% and an R_factor_ of 11.0% (Figure 1). The complex crystallized in spacegroup P2_1_2_1_2_1_ with one complete copy of the rhodesain mature catalytic domain (residues 1–215) in the asymmetric unit. The amino acid residues at the beginning of mature rhodesain structure are APAA, consistent with the predicted cleavage site at the N-termini of mature rhodesain, cruzain and other cathepsin L-like proteases. We recently reported the first crystal structure of rhodesain in complex with the vinyl sulfone inhibitor K11777 (PDB ID 2P7U) [36]. Superimposition of this vinyl sulfone complex with the K11002 complex reported here matches 214 α-carbons with root mean square distances of 0.27Å. An interesting feature of the K11002 complex is the observation of a dual conformation of the phenylsulfone moiety at the P1′ position of the inhibitor (Figure 2). The optimal model to data agreement was obtained by refinement of the two conformations as a 70%/30% combination of relative occupancies. We have previously observed that this group can flip out of the S1′ pocket [36].

The TbCatB•CA074 complex crystallized in spacegroup P2_1_ with two molecules in the asymmetric unit and was refined to 1.60Å resolution to a final R_free_ of 17.8% and R_factor_ of 14.7% (Figure 1). Chains A and B comprise residues 78–335 and 78–337 of full-length TbCatB, respectively. With the predicted maturation cleavage site of the enzyme between Pro93 and Leu94 [18], we were surprised to observe that our crystal structure of the activated, mature form of TbCatB contains an additional 16 residues at the N-terminus preceding the predicted site of activation. In light of the non-standard start of the catalytic domain, this structure is numbered from the N-terminal signal-sequence, with methionine as residue 1. For clarification in the text, TbCatB residues have the ‘standard’ mature domain numbering, according to human cathepsin B, in superscript. In the occluding loop of TbCatB (Pro189^105^-Phe213^126^) the electron density is somewhat diffuse in parts and these more flexible regions were therefore modeled at reduced occupancy. Interestingly, in chain B (where the electron density for the occluding loop is clear) we observed a dual conformation for the backbone carbonyl of His194^110^ and the backbone amide of His195^111^. A nearby electron density peak was modeled and refined as a water molecule. Although there was no significant difference density around this water when refined at full occupancy, we decided to model this position at 50% occupancy (HOH565) to reflect its transient interaction with the peptide chain in this region. Refinement of this peak as a common cellular/buffer ion at full occupancy (Li^+^, Na^+^, Mg^2+^, Ca^2+^) yielded significant positive or negative difference density.

### N-terminal sequence analyses of TbCat

In light of the unexpected residues observed N-terminal to the predicted maturation site, three separate aliquots of TbCatB were analyzed by Edman sequencing to determine, under the activation conditions described above, the full-length sequence of the mature domain prior to crystallization. All three reactions yielded LREAKRLNNV as the N-terminal peptide sequence. This corresponds to a peptide 61 residues into the full-length TbCatB sequence. The Asn at position 69 is Gly according to the published sequence (Genbank accession code AAR88085) and we presume this to simply be an experimental error due to the low amount of sample used for the blot. Additional N-terminal sequencing studies using E64 as the inhibitor and a larger quantity of sample confirm this residue to be Gly69 (data not shown). To rule out an activation event during cell culture and expression of the recombinant enzyme in *P. pastoris*, we also sequenced a purified, unactivated sample. This yielded the peptide EAEFALVAED; the first four residues are a cloning artifact from the 5′ end of the expression vector. The remaining six residues correspond to the expected beginning of the cloned sequence (minus the signal sequence). The implications of these findings are discussed below.

To determine whether an unexpected activation event occurs *in-vivo*, we compared the size, by Western Blot analysis, of our recombinant, purified mature TbCatB with a sample of the native protein present in crude *T. brucei* lysates (Figure S1). With the predicted size of mature TbCatB calculated to be 26–27kDa, the blot clearly shows that both recombinant and activated TbCatB are larger than predicted, with the recombinant form being the slightly larger of the two. While there are no modifications to prevent glycosylation of the native form, we have previously observed the size of the glycosylated and (Endo H-treated) deglycosylated protein to be the same (data not shown).

## Discussion

We report the crystal structures of rhodesain•K11002 and TbCatB•CA074, two papain family cysteine proteases implicated in the pathogenesis of *Trypanosoma brucei* infection (Figure 1). The structure of rhodesain•K11002 is similar to that of rhodesain•K11777 (PDB ID 2P7U) [36] with the bound inhibitor varying only at the P3 position (N-methyl piperazine in K11777, morpholino urea in K11002). While a number of hydrogen bonds are formed between residues lining the substrate-binding site and the inhibitor backbone, a number of hydrophobic residues also provide binding energy, principally in the S2 subsite (Figure 2), the subsite that confers selectivity for this class of enzyme. This is in contrast with the TbCatB•CA074 complex where hydrogen bonding between the enzyme and inhibitor dominate over hydrophobic interactions. The phenylsulfone moiety at P1′ is a common motif represented in many parasite cysteine protease•vinylsulfone complexes. The dual conformation of this moiety in the rhodesain•K11002 structure is unique for a parasite cysteine protease•vinylsulfone complex and we have not observed this in other high resolution structures of rhodesain or the closely related cruzain from *Trypanosoma cruzi*.

**Figure 3:**
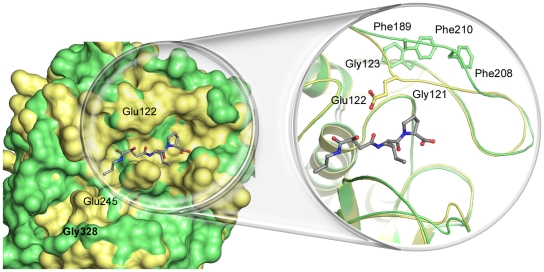
The substrate binding sites of TbCatB and mammalian CatBs. Surface and ribbon/ball and stick representations comparing the substrate binding sites of these two cysteine proteases. TbCatB (green), has a deep and spacious S2 pocket, with Gly328 at the bottom. Human CatB, a representative member of the mammalian homologs (1GMY - CA030 complex, yellow), has the larger Glu245 at this position and the pocket is shallower. In Human CatB•small molecule complexes, the occluding loop points into the substrate binding site. Conversely, the TbCatB occluding loop is pulled out of the active site. CA074 from the TbCatB crystal structure is shown in grey to orient the reader.

**Figure 4:**
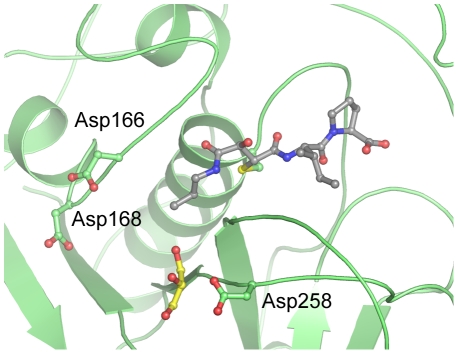
An acidic functionality around the S2 subsite of TbCatB. Ribbon/ball and stick illustrating an acidic functionality in the S2 subsite of TbCatB. CA074 is colored in grey and a bound molecule of glycerol (from the crystal cryosolution) is colored in yellow.

The TbCatB crystal structure, the first reported for this enzyme, is similar in overall structure to homologous cathepsins B-like enzymes studied (Table S1), with the majority of the variation found in the occluding loop region (discussed below). Our crystal structure also reveals several interesting features that are atypical of a cathepsin B-like cysteine protease. Cathepsin B family members were originally defined in vertebrate systems as possessing an acidic residue at the bottom of the S2 subsite that allows for the accommodation of basic residues in the pocket [37], [38]. TbCatB has a Gly at this position, which opens up the pocket allowing larger P2 substituents to be targeted to this part of the active site cleft (Figure 3). Homology modeling previously indicated an acidic functionality around the S2 subsite of TbCatB [39], that may be able to take advantage of a positive charge at the P2 position of small molecule inhibitors. These acidic residues line the sides (Asp166^75^, Asp168^77^, Asp258^175^) and bottom (Asp327^244^) of the pocket. In our structure Asp258^175^ and Asp327^244^ are available for binding and in each copy of TbCatB interact with a glycerol molecule from the cryoprotectant solution (Figure 4).

**Figure 5:**
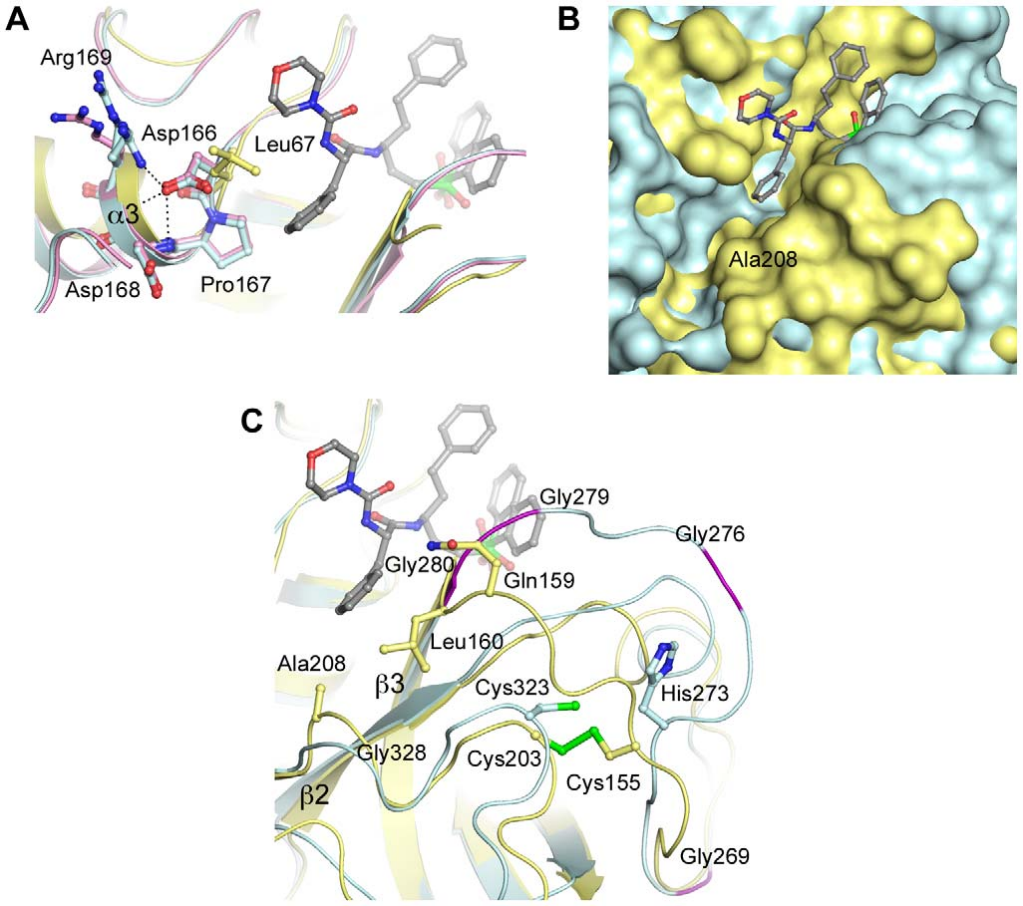
The S2 subsites of TbCatB and rhodesain. Superimposition of TbCatB•CA074 and rhodesain•K11002 reveals differences in the S2 subsites of the two enzymes. Rhodesain is colored yellow, TbCatB monomer A is blue and TbCatB monomer B (5A only) is pink. K11002 is included in grey to orient the reader w.r.t the active site. (A) The rhodesain pocket is partially restricted in comparison with TbCatB due to a Asp>Leu substitution (B) Ala208 at the bottom of the S2 subsite in rhodesain (Gly328 in TbCatB) makes the pocket shallower (C) The S2 subsite in TbCatB is further opened due to the conformation of the loop between strands β2 and β3. Glycine residues in the loop are colored purple.

Superimposition of TbCatB•CA074 and rhodesain•K11002 highlights structural differences that cause rhodesain to be more sterically restricted at the S2 subsite (Figure 5). Firstly, Asp166^75^ in TbCatB is substituted for a Leu in rhodesain (Leu67); Asp166^75^ in TbCatB is able to pack itself against helix α3 where it establishes a number of hydrogen bonding interactions (Figure 5a). Leu67 packs more favorably against the hydrophobic environment of the rhodesain S2 subsite with the large hydrophobic phenylalanyl at the P2 position of K11002. This residue therefore points in toward the substrate-binding site in rhodesain. Secondly, rhodesain has the larger Ala208 (Gly328^245^ in TbCatB) at the bottom of the S2 subsite, making the pocket shallower (Figure 5b). Finally, the loop between strands β2 and β3 in rhodesain is anchored to an adjacent loop (between strands β5 and β6) by a disulfide bridge between Cys155 and Cys203. A number of direct and water-mediated hydrogen bonding interactions stabilize this conformation and Gln159 and Leu160 are pulled into the S2 subsite to further narrow the pocket (Figure 5c). In TbCatB, the β2–β3 loop lacks the cysteine required to form the anchoring disulfide bridge, and is glycine-rich (Gly269^186^, Gly276^193^, Gly280^197^ and Gly281^198^) when compared with rhodesain. The additional flexibility allows the C-terminal portion of the TbCatB loop to adopt a conformation similar to that found in human cathepsin B, removed from the S2 subsite and oriented towards the prime sites. Of note, the mobility of this loop was recently alluded to in homology modeling studies by Mallari *et al.* in comparison with human cathepsin L [40].

TbCatB has an ‘occluding loop’, a unique feature of cathepsin B-like enzymes, which spans the prime side of the substrate binding site and distinguishes them from the cathepsin L-like enzymes [11], [21]. In TbCatB, the loop is three residues longer than in mammalian homologs and we note a dual peptide conformation between His 194^110^ and His195^111^. The occluding loop in TbCatB further deviates from homologous structures between residues 206^120^–210^123^. Human, rat and bovine cathepsin B have an invariant “GEGD” motif in this region. The glycine residues flanking Glu122 confer additional flexibility in this region such that the negatively charged residue is able to flip in and out of the active site [41] (Figure 3). The corresponding motif in TbCatB, “FNFD”, lacks this flexibility and both Phe208^121^ and Phe210^123^ stack with the N-terminal residue (Phe189^105^) of the occluding loop, creating a more stable opening around S1′. This feature of the TbCatB occluding loop presents the possibility to engineer additional specificity into inhibitors targeting this enzyme. Indeed Mallari *et al.* have shown that out of a series of 56 compounds, only those with a specific N9 substituent (hydroxypropyl) were reasonable human CatB inhibitors. The authors propose this may be due to the ability of this substituent to stabilize the flexible loop in a favorable conformation. This stabilizing interaction was not expected to be important in TbcatB; indeed TbcatB was tolerant of a wide range of substitutions at this position on the inhibitor scaffold.

**Figure 6:**
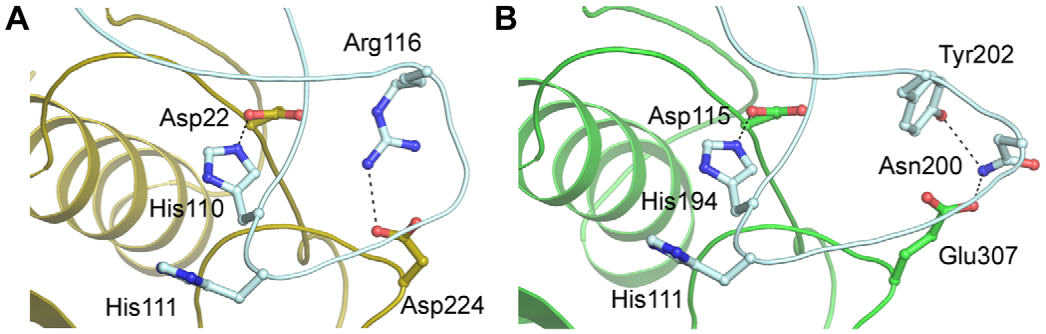
Interactions stabilizing the occluding loop in cathepsin B-like enzymes. Ribbon/ball and stick representations illustrating, important interactions that stabilize the occluding loop (pale cyan) of (A) Human cathepsin B (1GMY) and (B) TbCatB.

An interesting aspect of mammalian cathepsin B-like enzyme structure is the presence of two salt bridges (His110-Asp22 and Arg116-Asp224) that stabilize the “closed” conformation of the loop in the mature form (Figure 6). Mutations that disrupt either ion pair are correlated with a major increase in endopeptidase activity [42], presumably due to a corresponding increase in loop flexibility. While the His-Asp pair is conserved in TbCatB, Arg116 is substituted for Tyr202 and Asp224 is substituted for Glu307. In TbCatB, the acidic residue does not interact directly with Tyr202, but instead stabilizes the occluding loop at an insertion (relative to mammalian enzymes) through an interaction with Asn200 (Figure 6). It is tempting to speculate on the role that these substitutions might play, if any, on altering the characteristic pH dependance of cathepsin B activity/inhibition. However, at present, we have no biochemical evidence to support this assumption and clearly this is a point that requires further investigation through mutational analysis.

Cysteine proteases are expressed as inactive “zymogens” containing a “pro”-domain that aids in the proper folding of the full-length protein and suppresses the activity of the catalytic (mature) domain. Autoproteolysis results in cleavage between the pro and catalytic domains yielding the fully active, mature enzyme. Comparison of this TbCatB mature domain with crystal structures of the mature domains of mammalian cathepsin B enzymes, as well as the mature domains of rhodesain and papain, shows that the TbCatB structure has an unusually long N-terminus. However, further comparison with parasite cysteine proteases reveal that an elongated N-terminus is shared with the malarial proteases falcipain-2 (FP-2) and falcipain-3 (FP-3) [43], [44] (Figure S2A). Superimposition with TbCatB reveals the N-terminal extension of these proteases to be of similar length to that found in our structure (16 residues in falcipain-2 and 18 residues in falcipain-3). Furthermore, the extension in TbCatB establishes several polar and hydrophobic interactions with the L and R domains of the main α/β fold (Figure S2B), as is observed in structures of FP-2 and FP-3 (although this results in the malarial extensions adopting more extended secondary structure). While comparisons can be drawn between TbCatB and the malarial proteases, the atypical N-terminus of FP-2 and FP-3 was already identified before the structures were known, including the lack of a typical papain-family mature cleavage site [43]. Conversely, TbCatB does contain such a cleavage site and, contrary to our findings, residues upstream were expected to form part of the pro-domain. Our Edman sequencing data suggest the possibility of an even longer end (33 residues N-terminal to the predicted cleavage site ‘LPSS’). Analysis of the crystal packing in our TbCatB model suggests these residues may occupy a nearby solvent channel in the crystal and are therefore disordered. Alternatively, they may be lost during crystallization. Comparisons with the human and rat unactivated zymogens (PDB IDs 1MIR and 3PBH) show that, in the full-length “pro” form, the equivalent residues form a long loop and short helix that occlude the active site. The possibility of an additional 33 residues at the N-terminus of the mature TbCatB therefore remains an intriguing puzzle. While our sequencing data exclude the possibility of the recombinant enzyme being activated during yeast cell culture, we cannot exclude cellular activation of the endogenous enzyme as expressed by the native parasite. The Western Blot data show the latter to be larger upon activation than predicted by sequence analyses but slightly smaller than the recombinant form. We can only speculate that perhaps the native enzyme undergoes further processing during expression in *T. brucei*. Future experiments will be guided towards shedding further light on the unusual processing of this parasite cysteine protease.

## Supporting Information

Figure S1 Western Blot analysis of TbCatB. An immunoblot of native TbCatB from cultured parasites (Tb lysate, left) and recombinant, in-vitro activated TbCatB (tbcatB, right). The crude, unpurified Tb lysate shows two bands representing the zymogen (upper) and activated, mature (lower) forms. The purified recombinant protein sample contains only the mature form.
(0.22 MB TIF)

Figure S2 Superimposition of TbCatB, falcipain-2 and falcipain-3. (A) Comparison of the N-termini of TbCatB (red), falcipain-2 (light pink) and falcipain-3 (dark pink) in ribbon representation. The surface and other secondary structure belong to TbCatB and are colored as Figure 1. (B) Ribbon and ball and stick representations detailing interactions made between the N-terminus of TbCatB and the L and R domains of the enzyme. Colored as (A), with residues belonging to the N-terminus colored pink.
(3.56 MB TIF)

Table S1 Superimposition of TbCatB•CA074 with homologous cathepsin B•small molecule complexes.
(0.05 MB DOC)
