# Supplementary figures and images for: Crystal Structures of TbCatB and Rhodesain, Potential Chemotherapeutic Targets and Major Cysteine Proteases of Trypanosoma brucei
Source: PLoS Negl Trop Dis. 2010 Jun 8;4(6):e701. doi: 10.1371/journal.pntd.0000701 (PMC2882330; doi:10.1371/journal.pntd.0000701)

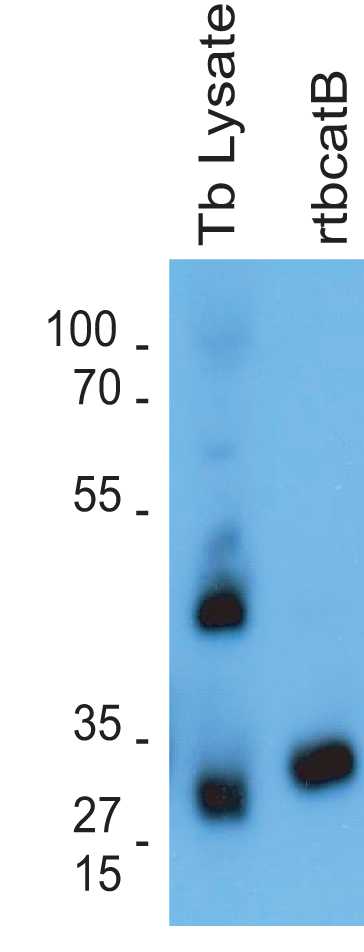

Supplement: Figure S1 — Western Blot analysis of TbCatB. An immunoblot of native TbCatB from cultured parasites (Tb lysate, left) and recombinant, in-vitro activated TbCatB (tbcatB, right). The crude, unpurified Tb lysate shows two bands representing the zymogen (upper) and activated, mature (lower) forms. The purified recombinant protein sample contains only the mature form. (0.22 MB TIF) [file pntd.0000701.s001.tif]

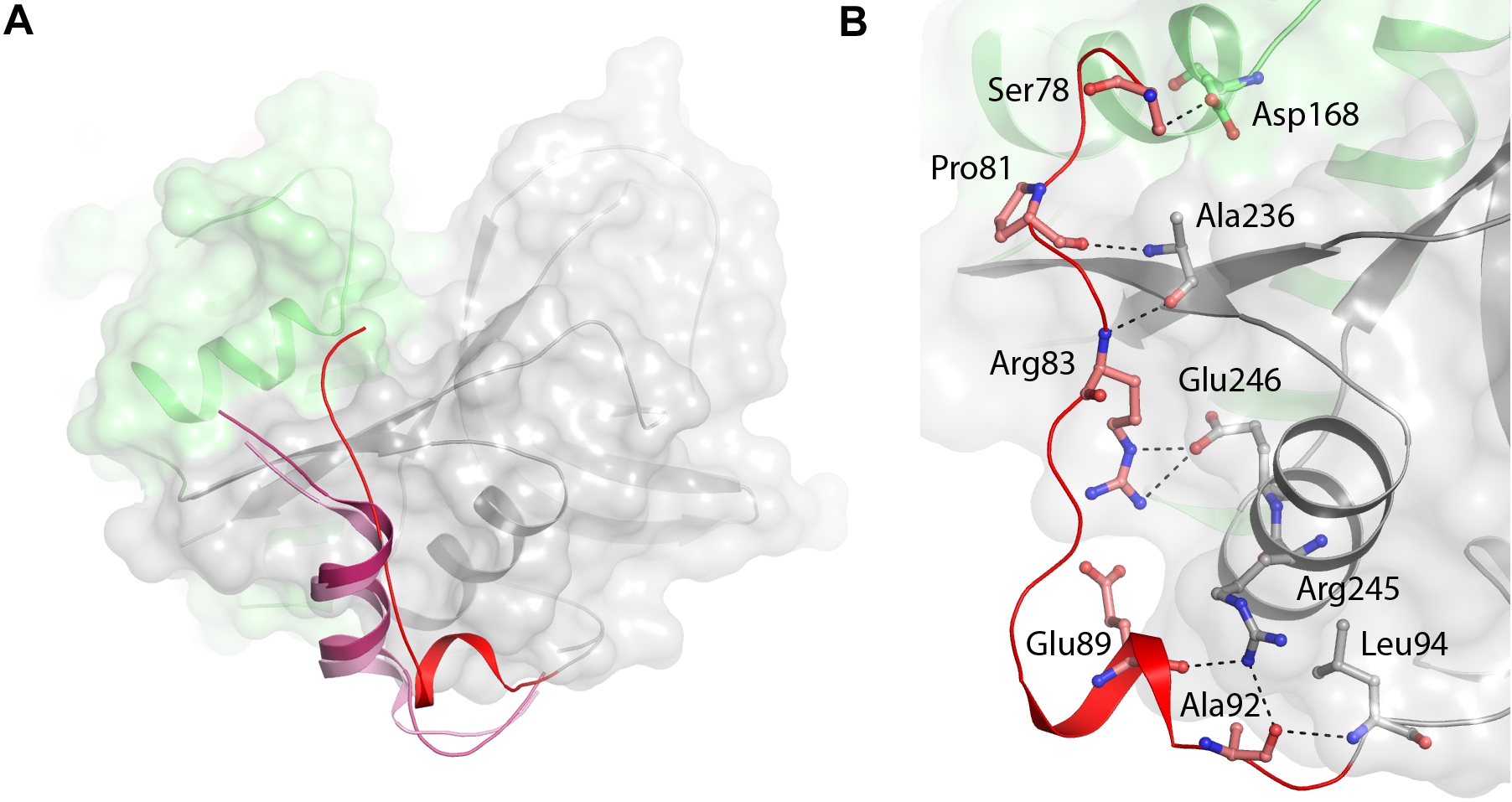

Supplement: Figure S2 — Superimposition of TbCatB, falcipain-2 and falcipain-3. (A) Comparison of the N-termini of TbCatB (red), falcipain-2 (light pink) and falcipain-3 (dark pink) in ribbon representation. The surface and other secondary structure belong to TbCatB and are colored as Figure 1. (B) Ribbon and ball and stick representations detailing interactions made between the N-terminus of TbCatB and the L and R domains of the enzyme. Colored as (A), with residues belonging to the N-terminus colored pink. (3.56 MB TIF) [file pntd.0000701.s002.tif]
