# Supplementary material for: Crystal Structures of TbCatB and Rhodesain, Potential Chemotherapeutic Targets and Major Cysteine Proteases of Trypanosoma brucei
Source: PLoS Negl Trop Dis. 2010 Jun 8;4(6):e701. doi: 10.1371/journal.pntd.0000701 (PMC2882330; doi:10.1371/journal.pntd.0000701)
Supplement: Table S1 — Superimposition of TbCatB•CA074 with homologous cathepsin B•small molecule complexes. (0.05 MB DOC) [file pntd.0000701.s003.doc]

**Table S1. Superimposition of TbCatB•CA074 with homologous cathepsin B•small molecule complexes**

| Structure•small molecule (PDB ID)^1^ | Matching Cα positions | rmsd (Å) |
| --- | --- | --- |
| Bovine•CA042 (2DC7) | 212 | 0.73 |
| Bovine•CA059 (2DC8) | 231 | 0.87 |
| Bovine•CA073 (2DC6) | 230 | 0.82 |
| Bovine•CA074 (1QDQ) | 218 | 0.80 |
| Bovine•CA074Me (2DC9) | 213 | 0.72 |
| Bovine•CA075 (2DCA) | 218 | 0.77 |
| Bovine•CA076 (2DCB) | 222 | 0.80 |
| Bovine•CA077 (2DCC) | 229 | 0.81 |
| Bovine•CA078 (2DCD) | 231 | 0.84 |
| Bovine•E64c (1ITO) | 229 | 0.82 |
| Human•dipeptidyl nitrile (1GMY, A) | 229 | 0.85 |
| Human•dipeptidyl nitrile (1GMY, B) | 228 | 0.81 |
| Human•dipeptidyl nitrile (1GMY, C) | 229 | 0.85 |
| Rat•chloromethylketone inhibitor (1THE, A) | 230 | 0.90 |
| Rat• chloromethylketone inhibitor (1THE, B) | 229 | 0.77 |
| Rat•2-pyridinethiol (1CTE, A) | 226 | 0.77 |
| Rat•2-pyridinethiol (1CTE, B) | 230 | 0.85 |

^1^ Superimpositions performed with TbCatB monomer B
